# Successful conservative medical management of an interstitial ectopic pregnancy at 10 weeks of gestation: A case report

**DOI:** 10.1016/j.crwh.2020.e00284

**Published:** 2020-12-26

**Authors:** A. Galani, A. Zikopoulos, E. Moulias, M. Paschopoulos, K. Zikopoulos

**Affiliations:** a University Hospital of Ioannina, Department of Obstetrics and Gynecology, Ioannina, Greece; b Obstetrics and Gynecology Royal Cornwall Hospital, Cornwall, UK

**Keywords:** Interstitial pregnancy, Conservative management, Ectopic pregnancy

## Abstract

**Background:**

Interstitial pregnancy is a rare type of ectopic pregnancy, accounting for 2–6% of ectopic pregnancies, but it can be life threatening. There is no clear consensus on management, either surgical or medical, and it depends on hemodynamic stability and whether fertility-sparing treatment is requested.

**Case Presentation:**

We present the case of a 35-year-old woman (G2, P1) who was diagnosed with an interstitial pregnancy at 10 weeks of gestation following in vitro fertilization. She was hemodynamically stable and requested fertility-sparing treatment. She was managed successfully with methotrexate and folinic acid with a hospital stay of 17 days.

**Conclusion:**

Interstitial pregnancy can be managed medically. However, these patients require close monitoring.

## Highlights

• Iterstitial ectopic pregnancy accounts for 2-6% of ectopic pregnancies.
• Management may be either surgical or medical.
• Successful medical management with methotrexate regimen of a 10-week interstital pregnancy is described.

## 1 Introduction

Interstitial pregnancy is a challenging diagnosis and, if missed, can be life threatening. This type of ectopic pregnancy is rare, accounting for 2–6% of ectopic pregnancies, and risk is increased in the presence of pelvic pathology (pelvic inflammatory disease, endometriosis, leiomyomas), previous ectopic pregnancy, tubal surgery and in vitro fertilization [[1], [2], [3], [4], [5]]. An interstitial pregnancy occurs when implantation occurs in the most proximal section of the fallopian tube (called the interstitial portion), which is within the myometrium. The potential to cause life-threatening hemorrhage results in a 2–5% mortality rate. [5].

The treatment of an interstitial pregnancy is controversial and there is no clear consensus regarding optimal management [3,5]. Traditional surgical management included wedge resection by laparoscopic/open surgery or hysterectomy [4,5]. More recently, less invasive approaches, such as laparoscopic cornuostomy, have been developed [5,6]. In addition, local methotrexate injection can be administered during laparoscopy or with ultrasound guidance. Conservative medical management usually refers to the administration of intravenous or intramuscular methotrexate and it is usually preferred when fetal cardiac activity is not present and the serum β-HCG level is low (<10.000 IU/l) [4].

## 2 Case Presentation

A 35-year-old Caucasian pregnant woman (G2, P1) presented at the antenatal department for a routine ultrasound scan following a positive pregnancy test. She was10 weeks and 4 days pregnant after in vitro fertilization (IVF). This was her second pregnancy. Her first pregnancy was delivered two years previously by caesarean section for breech presentation. Both of the pregnancies were achieved through IVF. During her caesarean section she had experienced pulmonary aspiration.

**Fig. 1:**
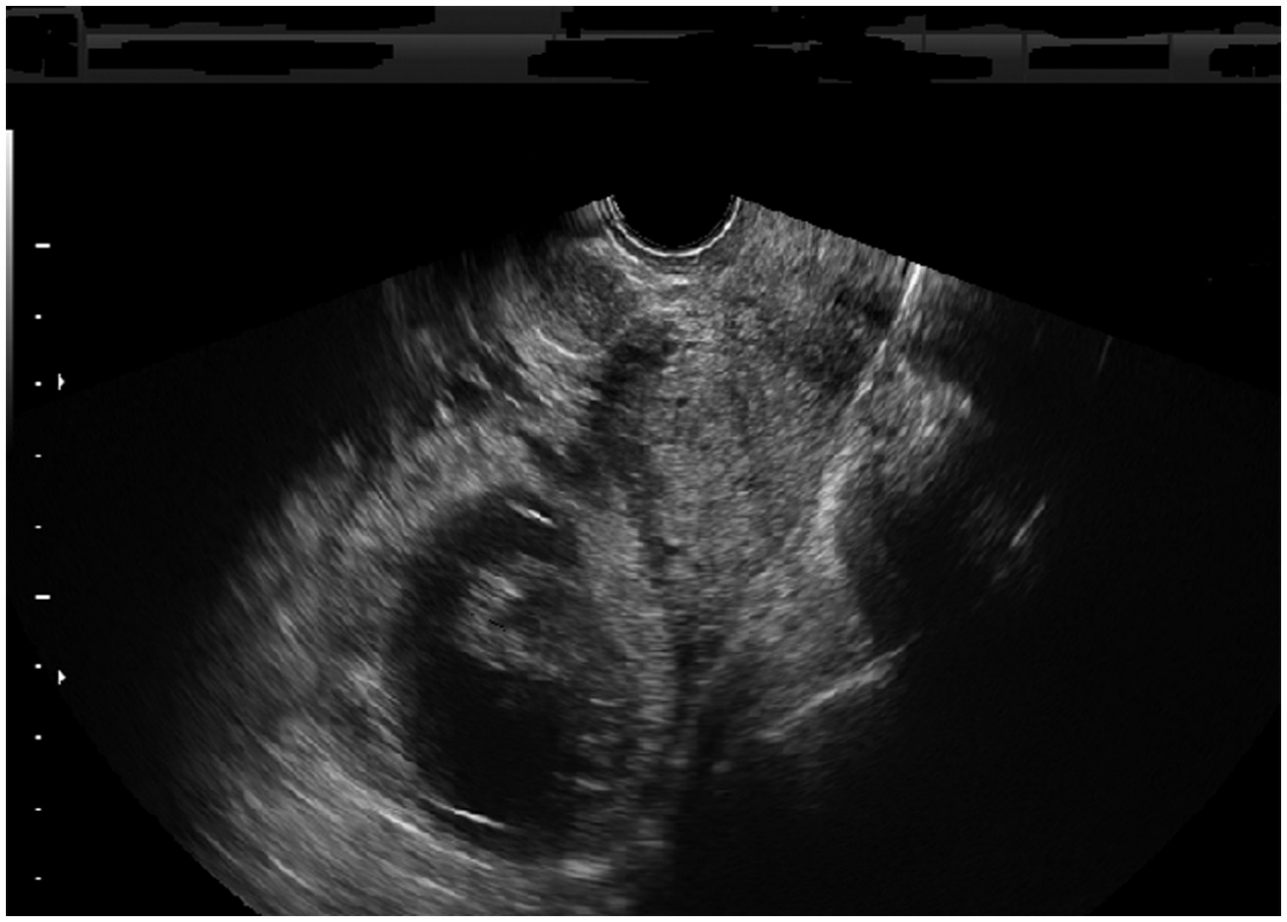
The gestational sac at the right uterine fundus surrounded by myometrium.

**Fig. 2:**
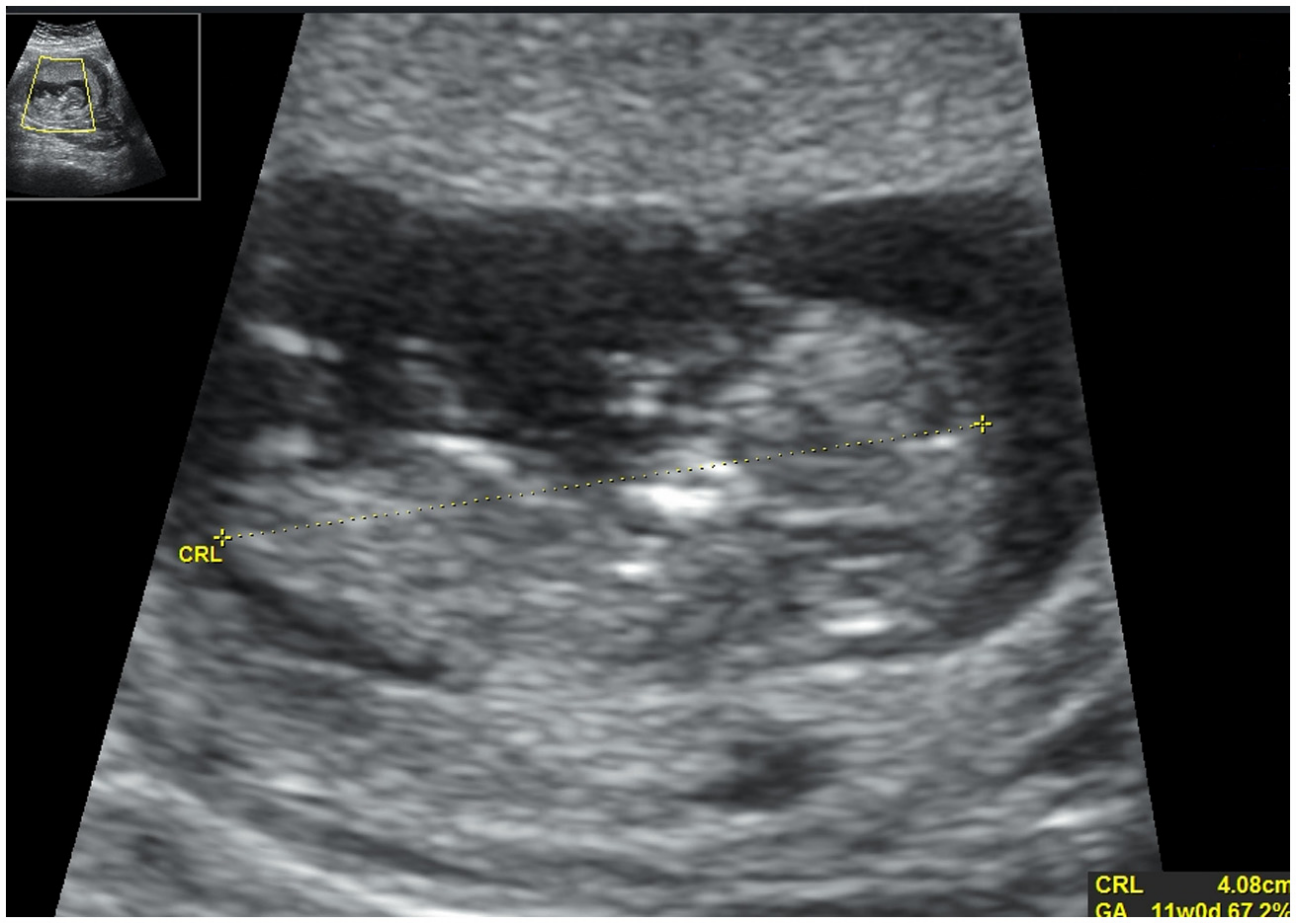
A CRL of 40.8 mm was measured during the first scan.

Her ultrasound scan findings were reported as follows: A gestational sac with regular margin was seen at the right uterine fundus surrounded by myometrium (Fig. 1). Fetal pole was detectable, crown–rump length (CRL) was 40.8 mm (Fig. 2) and fetal heart was positive. The patient was stable and she denied any discomfort, pain or vaginal bleeding. Her blood investigations on admission were as follows: Hb 13.1 g/dl; β-HCG 60530 IU/l; WBC 12430 10^3/μl.

The diagnosis was discussed with the patient and the potential treatment was explained to her, emphasizing that, given her scan findings and blood results, a surgical approach, especially laparoscopic cornuostomy, might be more appropriate. The patient expressed her wish to preserve fertility and was reluctant to have surgery unless she became hemodynamically unstable because of her previous pulmonary aspiration. Therefore, conservative medical treatment was commenced. Methotrexate 100 mg i.v. was administered to the patient on day 1 of hospitalization, followed by folinic acid 10 mg i.m. the next day.

**Fig. 3:**
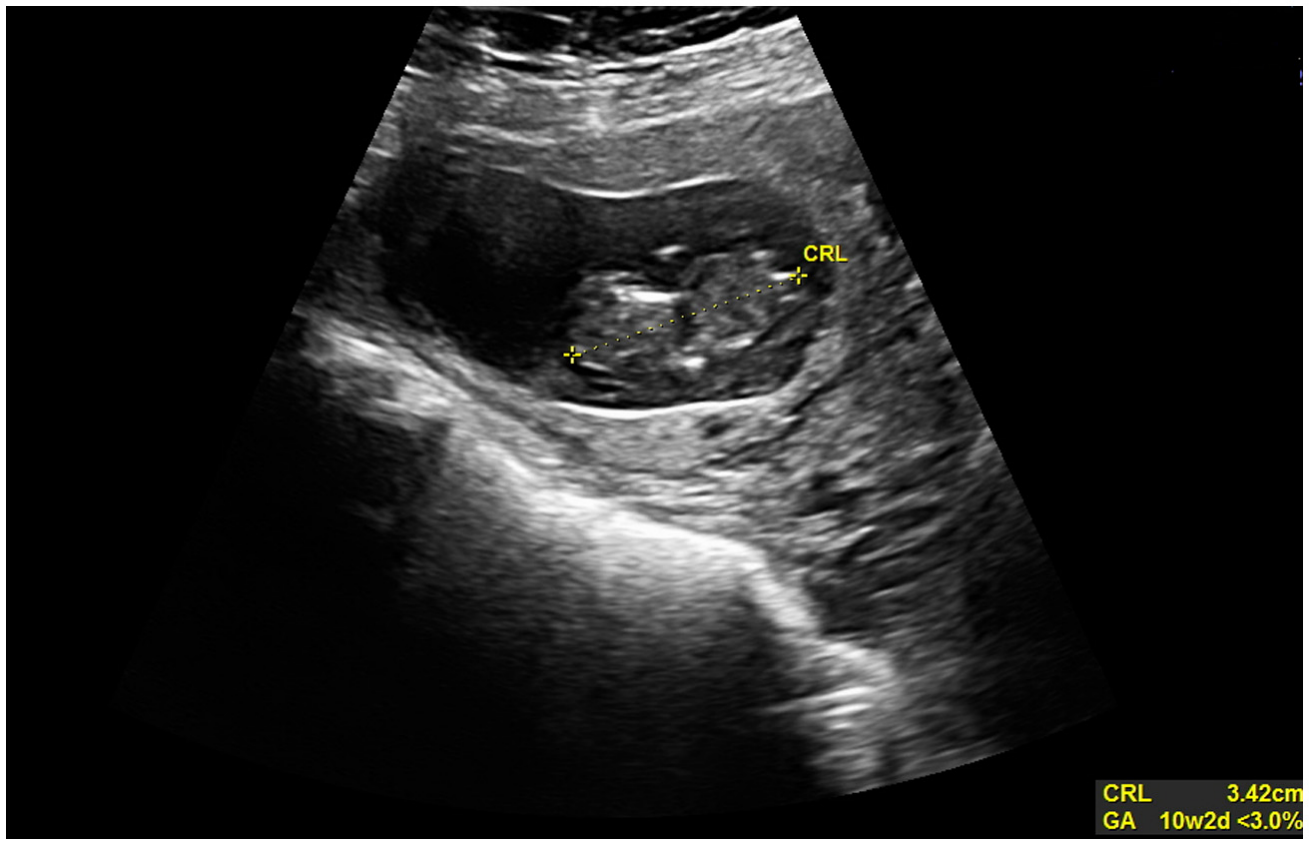
CRL of 34.2 mm after methotrexate injection into the gestational sac.

On day 3 of hospitalization, blood investigations were repeated and the results were as follows: β-HCG 65953 IU/l; Hb 13.0 g/dl. The patient underwent a new ultrasound scan on the same day. During this scan, the fetal heart was undetectable. The new findings were discussed with the patient and an operation was suggested; however, she was still reluctant to have surgery. On day 4 of hospitalization, direct methotrexate injection into the gestational sac was performed under ultrasound guidance [2]. Three days after the methotrexate injection a new ultrasound scan was performed and the CRL measurement was seen to have decreased to 34.2 mm and (Fig. 3).

**Fig. 4:**
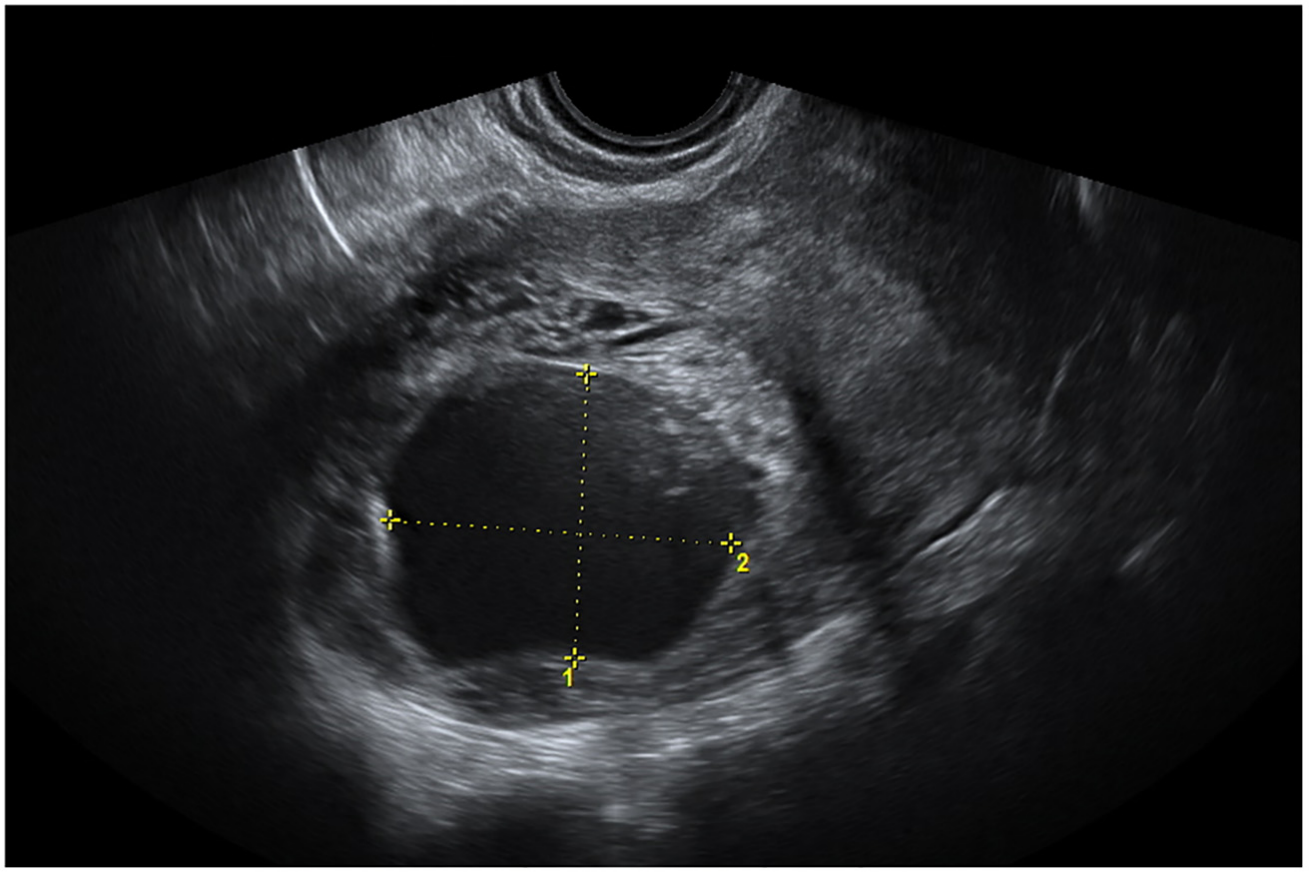
No detectable fetal pole into the gestational sac, 3 weeks after discharge.

**Fig. 5:**
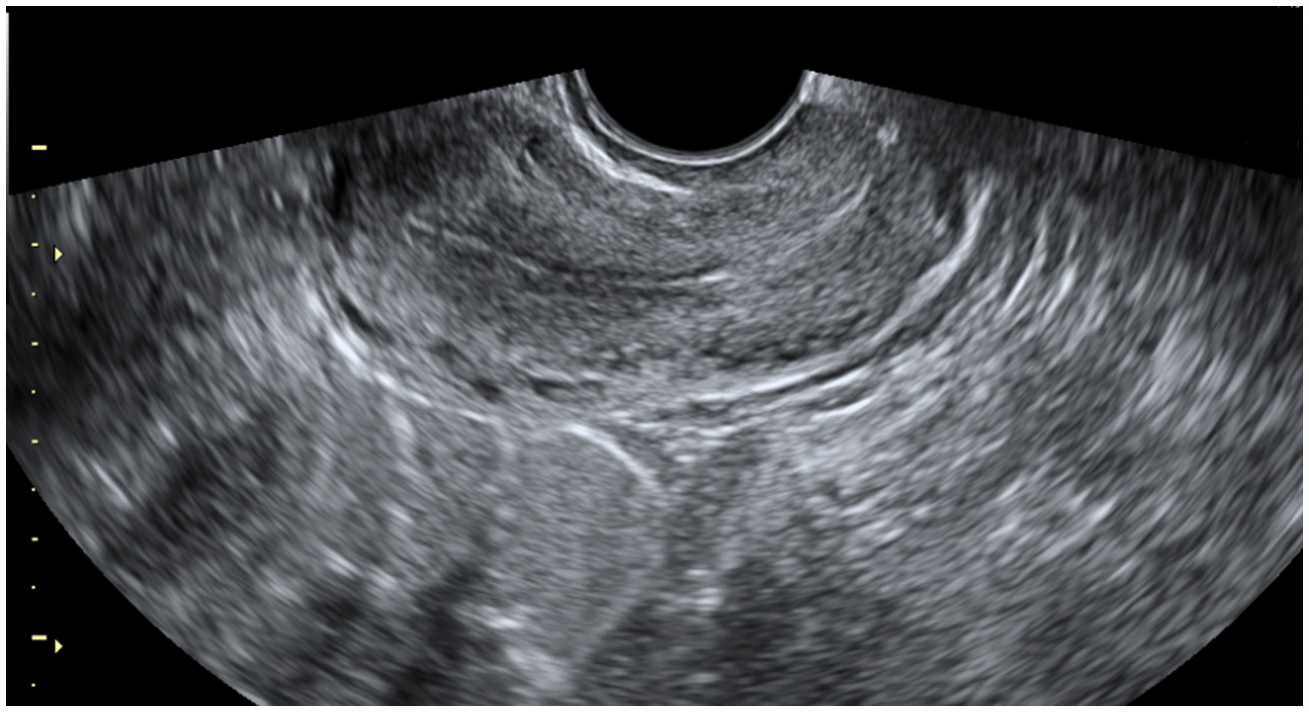
Uterus at the last follow-up 6-week visit.

The patient was hospitalized for 17 days in total and was given the methotrexate/folinic acid regimen twice more. During her conservative medical treatment the patient was stable and had no discomfort, vaginal bleeding or any drug side-effects. On day 16 of hospitalization β-HCG was 2051 IU/l and on the repeated scan the fetal pole was 28 mm. She was discharged on day 17 and she was asked to return for follow-up after 3 weeks. On follow-up, her β-HCG was 60.57 IU/l and her scan findings were reassuring as no fetal pole was detectable (Fig. 4). She was asked to return in 6 weeks for further follow-up. At her last follow-up visit, the uterus appeared to be normal and no gestational sac was seen (Fig. 5) and β-HCG was 9.3 IU/l.

## 3 Discussion

Interstitial pregnancy is a rare type of ectopic pregnancy, accounting for 2–6% of all ectopic pregnancies, that comes with a high mortality rate [5,[7], [8], [9]].

The diagnosis and the management are challenging and it can be easily misdiagnosed. The traditional approach is surgical, but expectant or medical management can be offered to women who are hemodynamically stable and have no obvious risk of immediate rupture [5,10]. The main disadvantages of systemic methotrexate include higher drug dosage, more systemic side-effects and lower tissue concentration. Another concern is recurrence of ectopic pregnancy on the same side [1].

Medical management requires close monitoring of the patient [4,5,7]. This patient was hospitalized for 17 days in total. Inpatient management was preferred to outpatient follow-up as the patient lived almost 2 h away from the hospital.

## 4 Conclusion

The methotrexate/folinic acid regimen can be an effective and reasonable option in interstitial pregnancy management, especially when fertility preservation is desirable. Close monitoring and ensuring that the patient is stable are key to successful conservative medical management. During treatment, repeated blood investigations and ultrasound scans are recommended.

In this particular case, the combination of local and systemic methotrexate resulted in a good outcome.
